# Combination of the effect of ginger and anti‐inflammatory diet on children with obesity with nonalcoholic fatty liver disease: A randomized clinical trial

**DOI:** 10.1002/fsn3.3218

**Published:** 2023-01-24

**Authors:** Negin Kamari, Mehdi Moradinazar, Mahmoud Qasemi, Tina Khosravy, Mehnoosh Samadi, Hadi Abdolahzad

**Affiliations:** ^1^ Nutritional Sciences Department, School of Nutrition Sciences and Food Technology Kermanshah University of Medical Sciences Kermanshah Iran; ^2^ Behavioral Disease Research Center Kermanshah University of Medical Sciences Kermanshah Iran; ^3^ Department of Pediatrics, School of Medicine Kermanshah University of Medical Sciences Kermanshah Iran; ^4^ Department of Health Nutrition Lorestan University of Medical Sciences Khoram‐Abad Lorestan Iran; ^5^ Research Center for Environmental Determinants of Health (RCEDH), School of Public Health Kermanshah University of Medical Sciences Kermanshah Iran

**Keywords:** anti‐inflammatory diet, ginger, nonalcoholic fatty liver, obesity

## Abstract

Nonalcoholic fatty liver disease (NAFLD) is the most common cause of liver disease in children. Following earlier reports on an increase in the prevalence of childhood obesity, NAFLD is now becoming increasingly common in children. Although no definitive cure exists, early management, early diagnosis, and treatment can reduce its complications. This study aims to determine the effectiveness of the combination of ginger and an anti‐inflammatory diet (AID) in children with obesity on fatty liver management. This randomized clinical trial was conducted on 160 children with obesity aged 8–11 years, with a mean (SD) weight of 65.01 (9.67) kg, mean (SD) height of 139.87 (7.37) cm, and mean (SD) body mass index of 33.40 (5.59) kg/m^2^. The study duration was 12 weeks. Children were divided into four groups: ginger (G), AID, ginger plus an AID (GPA), and control. Ginger capsules comprised 1000 mg of ginger, whereas the AID comprised fruits and vegetables, fish, turkey, and chicken (without skin) with lean meat, omega‐3 sources, nuts, legumes, probiotic products, and elimination of inflammatory food. Following the intervention, serum fasting blood sugar and high‐sensitivity C‐reactive protein levels were significantly decreased in the AID (*p* = .006 and .002, respectively), G (*p* = .04 and <.001, respectively), and GPA (*p* <.001 in both cases, respectively) groups. Further, in the G and GPA groups, there was a significant decrease in body mass index (*p* = .04 in both cases, respectively), waist circumference (*p* = .009 and .003, respectively), waist‐to‐height ratio (*p* = .02 and .005, respectively), alanine aminotransferase (*p* = .004 and <.001, respectively), total cholesterol (*p* = .0002 and .0001, respectively) and low‐density lipoprotein‐cholesterol (*p* < .001 and <.001, respectively). Eventually, serum aspartate aminotransferase was decreased (*p* < .001) and high‐density lipoprotein‐cholesterol (*p* = .03) was increased significantly in the GPA group. As a main finding of this study, hepatic steatosis significantly decreased in the G and GPA groups. Ginger supplementation can effectively improve NAFLD in children, and its effectiveness was further increased when combined with an AID.

## Graphical abstract

Ginger as a nonmedicinal supplement can improve fatty liver in children with obesity, while adherence to an anti‐inflammatory diet will increase its overall effectiveness. This work is part of Negin Kamari's MSc thesis on children aged 8–11 with fatty liver. We investigated the effectiveness of ginger and an anti‐inflammatory diet after a 12‐week intervention period.

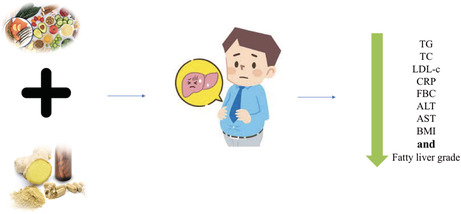

## 1 INTRODUCTION

Nonalcoholic fatty liver disease (NAFLD), known as fatty liver disease associated with metabolic dysfunction, is the buildup of extra fat in liver cells that is not caused by alcohol (Stefan, Häring, & Cusi et al., 2019). Fatty liver disease is of two types: NAFLD and nonalcoholic steatohepatitis, with the latter also including inflammation of the liver (Chalasani et al., 2018; Lazarus et al., 2021). NAFLD includes a wide range of liver disorders from hepatocellular steatosis to more severe nonalcoholic steatohepatitis, which may progress to hepatic fibrosis and cirrhosis (Marjot et al., 2020). NAFLD is present in approximately 38% of children with obesity worldwide (Draijer et al., 2019). Its prevalence in Iran is reported to vary between 42.6% and 77.1%. Fatty liver is expected to be the most common cause of chronic liver disease in children (Papandreou et al., 2012; Li et al., 2021; Chan et al., 2004). This is particularly interesting, as emerging NAFLD data associate children with an increased risk of adult morbidity and mortality, as well as cardiovascular disease and type 2 diabetes (Draijer et al., 2019).

Diet appears to be a risk factor for pediatric NAFLD (Fitzpatrick & Dhawan, 2019). However, it remains unclear which components of the diet affect the growth and development of NAFLD in children (Gibson et al., 2015). Based on epidemiological data, children with obesity with NAFLD have a higher intake of fructose, saturated fat, and less fiber than children without NAFLD (Fitzpatrick & Dhawan, 2019; Vancells Lujan, Viñas Esmel & Sacanella Meseguer, 2021). Regarding dietary models, in a population cohort study of adolescents, strict adherence to a Western diet at 14 years of age was correlated with an increased risk of NAFLD at 17 years. This association focused on body mass index (BMI; Oddy et al., 2013).

As there is no Food and Drug Administration (FDA)–approved medical treatment to treat pediatric NAFLD (Rinella et al., 2019), treatment protocols focus on lifestyle modifications to reduce disease‐related risk factors (Draijer et al., 2019). Standard recommendations for children with overweight and obesity indicate weight management and physical activity (Vos et al., 2017; Chalasani et al., 2018). Previous studies in adults have shown that a diet rich in antioxidants and anti‐inflammatory compounds can effectively treat NAFLD (Eslamparast et al., 2015). Furthermore, evidence has recommended the use of certain functional foods and nutraceuticals as potential therapeutic agents for NAFLD, as they may disrupt signaling pathways in the pathogenesis of NAFLD (Goodarzi et al., 2019).

Ginger (*Zingiber officinale*) is a flowering plant whose rhizome (ginger root or ginger) is widely used as a spice and as a popular medicine owing to its various pharmacological (as an immunoregulator and tumorigenesis inhibitor), anti‐inflammatory, antiapoptotic, and antiemetic effects. Most of these medicinal effects of ginger are related to various compounds such as gingerol and shogaol. More than 40 antioxidants have been identified in ginger. The FDA has confirmed that ginger is a dietary supplement (Suman et al., 2021). Previous studies have linked the anti‐diabetes, anti‐cancer, and anti‐inflammatory effects of ginger to its active ingredient (Thomson et al., 2002; Bhandari, Kanojia & Pillai, 2005). Ginger extract has been shown to exhibit antioxidant activity and reduce proinflammatory biomarkers (Grzanna, Lindmark & Frondoza, 2005). In addition, recent studies in patients with type II diabetes and hyperlipidemia have shown that ginger can reduce insulin resistance and serum triglyceride (TG) concentrations (Arablou et al., 2014; Shidfar et al., 2015).

Accordingly, the hypothesis of this study is that ginger supplementation and an anti‐inflammatory diet (AID) can be introduced as a new therapeutic strategy for NAFLD. A randomized, double‐blind study was designed to test this hypothesis by considering the effects of anti‐inflammatory dietary compounds in controlling the progression of fatty liver and the role of ginger as an antioxidant. Therefore, this study aims to determine the combined effect of ginger and an AID on lipid profiles, blood glucose, liver enzymes, inflammatory markers, and liver fat status in children with overweight with NAFLD.

## 2 METHODS AND MATERIALS

### 2.1 Participants and study design

Patients were identified and recruited from Mohammad Kermanshahi Hospital, Iran. Admission criteria for selection of included patients were as follows (Ashtary‐Larky et al., 2018): BMI >85th percentile, normal upper levels of alanine aminotransferase (ALT) >1.5 times the upper limit of the normal range, ultrasonography (indicated fat aggregation; grades 1–3), age 8–11 years, and not on weight loss diets. Exclusion criteria were a history of disease such as diabetes, cardiovascular, liver, and kidney disease; receiving supplements in the last 6 months; under hormone therapy; using not more than 10% of the capsules given in each follow‐up; and unwillingness to continue the study protocol. In total, 160 children met our inclusion criteria and were selected for this study. To control for the confounding factor, a stratification approach was applied. First, patients were divided into two groups of girls and boys and then based on the amount of physical activity they were divided into three groups: low (<15 points from questionnaire), medium (15 points from questionnaire), and severe (>15 points from questionnaire). In the next step, samples in each group were divided into two categories based on calorie consumed from macronutrient intake: low (<2000 Kcal/day) and high (>2000 Kcal/day). Finally by simple randomization, samples were divided into four groups of intervention (three groups were randomly selected for the intervention group: *n* = 40 for GPA, G, and AID, respectively) and a control group (*n* = 40). For the group receiving the AID, we recommended consumption of anti‐inflammatory foods, including poultry, fish, omega‐3 sources, colored fruits and vegetables, nuts and legumes, probiotic, and low‐fat products, with elimination of inflammatory foods such as high‐fat foods, simple sugars, fast food, processed meats (e.g. sausages, hot dogs), chips, crackers, and soft drink, for 12 weeks. Ginger powder supplement used in this study is a ready‐made product. Ginger (*Z. officinale*) capsules contained 1000‐mg ginger rhizome powder. A placebo containing wheat flour (1000 mg) was prepared in the same form and color as a ginger supplement in the Pharmacy Faculty Lab of Kermanshah University of Medical Sciences.

### 2.2 Dietary intake and supplementation

We matched sex, physical activity level, and calorie intake in the four groups to control for confounding factors. To check AID compliance, participants complete 24‐hour dietary recall weekly. A 3‐day food record questionnaire (1 day off and 2 days non‐off) was administered to other groups at the beginning and end of the study. The intakes of energy, macronutrients, micronutrients, and water were measured using the Modified Nutritionist IV Software (version 3.5.2, First Data‐Bank; Hearst Corp., San Bruno, CA). The physical activity level of patients was evaluated using the BEACK Questionnaire (Baecke et al., 1982) at the beginning and end of the study. This questionnaire is a tool that evaluates individual's habitual physical activities over the pervious 12 months. It should be noted that the dosage of ginger used and the duration of the intervention have been determined according to previous studies (Rafie et al., 2020). For accuracy of double‐blindness, capsules were coded by a person other than the study researchers, as A or B. To calculate the compliance rate of patients to the supplementation, all patients received supplementation for 4 weeks and were asked to deliver the cans of capsules at each visit, and then they were given supplements for the next 4 weeks. To control adherence to the AID, patients complete a 24‐h recall (weekly consumption data). Patients were also followed up on a weekly basis by telephone to be aware of possible side effects and to ensure the use of supplements.

### 2.3 Clinical and para clinical assessments

According to the WHO definition, the anthropometric measurement of the patients’ height, weight, waist circumference (WC), and hip circumference was performed at the beginning and end of the intervention. BMI was calculated by dividing weight by squared height, WHR by dividing waist to hip circumference, and waist‐to‐height ratio (WHtR) by dividing waist by height (Ebrahimzadeh Attari et al., 2017). Blood (10 cc) was taken from all participants at the beginning of the study and the end of the 12th week after a 10–12‐h fasting. Samples taken were centrifuged, and their serum was separated. For further tests, the serum was stored at −80°C. The serum TG level was measured using the enzymatic calorimetric method, while the total cholesterol (TC), high‐density lipoprotein (HDL), and low‐density lipoprotein (LDL) of plasma were measured by the photometric enzymatic method. The serum levels of liver enzymes, including alkaline phosphatase (ALP), ALT, and aspartate aminotransferase (AST), were measured by colorimetry (Parsazmun Co). Fasting blood sugar (FBS) was measured using the glucose oxidase method (Pars Azmoon Co). High‐sensitivity C‐reactive protein (hs‐CRP) was determined using LDN Labor Diagnostics Nord GmbH & Co. KG (Nordhorn, Germany) kits.

### 2.4 Liver ultrasound

Liver ultrasound was performed by the same experienced radiologist using an Acuson Sequoia C512 scanner equipped with a 15 L8 transducer (Universal Diagnostic Solutions). Normal liver, absent steatosis (grade 0) was defined as having a normal liver echo texture; mild steatosis (grade 1) as slight and diffuse increase in fine parenchymal echoes with normal visualization of the diaphragm and portal vein borders; moderate steatosis (grade 2) was defined as moderate and diffuse increase in fine echoes with slightly impaired visualization of the diaphragm and portal vein borders; and severe steatosis (grade 3) was defined as fine echoes with poor or no visualization of the diaphragm, portal vein borders, and posterior portion of the right lobe.

### 2.5 Statistical analysis

Statistical analysis was performed using STATA software version 14.2. Paired *t*‐test and one‐way analysis of variance were used to compare quantitative data and χ^2^ test was used to compare qualitative data. Significance level was considered when *p* is <.05.

## 3 RESULT

### 3.1 Patient inclusion and safety

**FIGURE 1:**
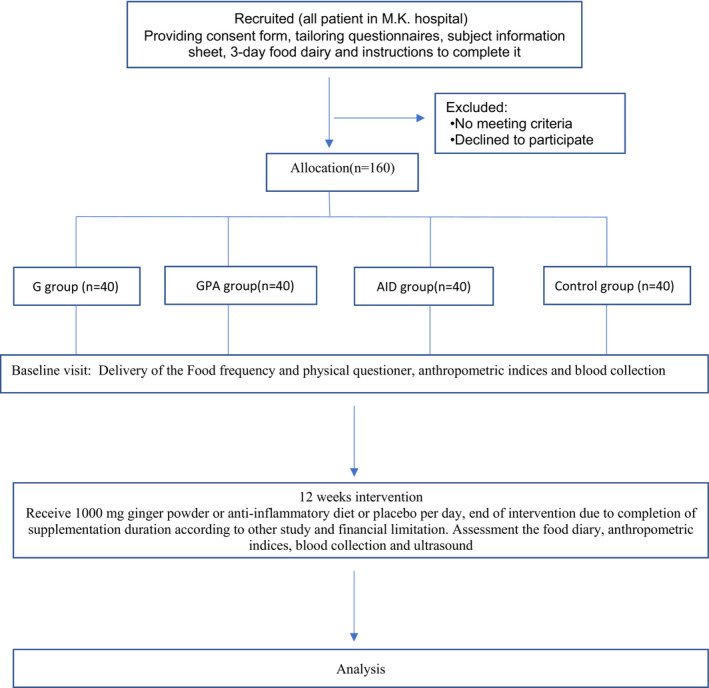
Flowchart of the study. AID, anti‐inflammatory diet; G, ginger; GPA, ginger plus anti‐inflammatory diet

According to the flowchart in Figure 1, 40 patients in the control group and 40 patients in each intervention group completed the study. None of the patients showed any allergic reaction or side effects of supplementation.

### 3.2 Participants’ characteristics

A total of 160 patients were included in the study and were randomly assigned to one of the four groups (*n* = 40). All patients completed study and their data entered into the final analysis. Demographic characteristics are summarized in Table 1. There was no statistically significant difference between the four groups at the beginning of the study.

**TABLE 1:** Baseline characteristics in the four groups

Variable/group		Control	AID	G	GPA	*p* Value
Gender (boy/girl) (%)		50/50	50/50	50/50	50/50	>.99
Age^a^ (y)		9.5 ± 1.08	9.5 ± 1.27	9.3 ± 1.07	9.4 ± 1.03	.6
Birth weight^a^ (g)		3272 ± 717	3234 ± 399	3195 ± 455	3260 ± 618	.77
Average time watching TV^a^ (h)		3 ± 2.5	3.5 ± 2.9	3 ± 1.5	3 ± 1	.12
Average time for computer games^a^ (h)		1.1 ± 0.86	1 ± 0.79	1.2 ± 0.56	1 ± 0.69	.90
Maternal age at delivery^a^ (y)		28 ± 5.4	28.3 ± 6.2	28.7 ± 5.1	27.8 ± 5.4	.59
Parents’ education^b^	Diploma or subdiploma	25.2 (28)	36.7 (24)	38 (5)	0 (0)	.188
	Postdiploma or bachelor	27.6 (20)	0 (0)	35.6 (12)	36.7 (13)	
	Master or PhD	32.3 (3)	25.1 (10)	24.5 (11)	18 (5)	
Family history of obesity^b^	None	15 (9)	27.9 (6)	23.6 (1)	33.4 (12)	.151
	Paternal family	27 (6)	43.1 (1)	20.7 (13)	9.1 (5)	
	Maternal family	23.2 (12)	24.1 (11)	22.7 (5)	29.9 (1)	
	Both	25.1 (11)	24.9 (3)	25.4 (12)	24.5 (9)	
Physical activity^a^ (points)		31.1 ± 3.2	31.1 ± 3.9	31.1 ± 4	31.1 ± 3	.97

*Note*: Data are expressed as mean ± standard deviation or *n* (%). Data are tested by ^a^one‐way analysis of variance and ^b^chi‐square test.

Abbreviations: AID, anti‐inflammatory diet; G, ginger; GPA, ginger plus anti‐inflammatory diet.

### 3.3 Primary outcome

#### 3.3.1 Biochemical assessment

**FIGURE 2:**
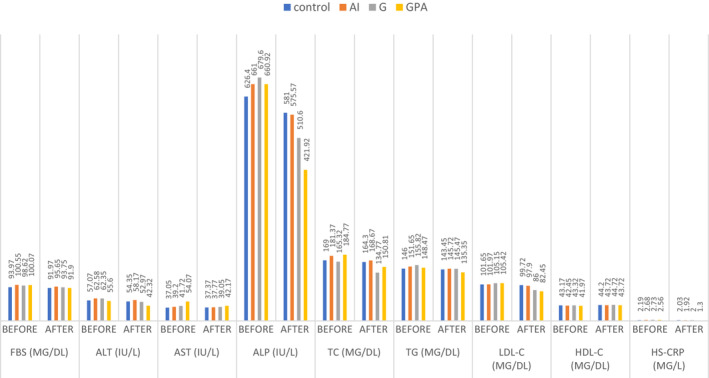
Intra‐ and intergroup comparisons of the changes from baseline to the end of the intervention for biochemical parameters. AID, anti‐inflammatory diet; ALP, alkaline phosphatase; ALT, alanine aminotransferase; AST, aspartate aminotransferase; FBS, fasting blood sugar; G, ginger; GPA, ginger plus anti‐inflammatory diet; HDL‐c, high‐density lipoprotein‐cholesterol; hs‐CRP, high‐sensitivity C‐reactive protein; LDL‐c, low‐density lipoprotein‐cholesterol; TC, total cholesterol; TG, triglyceride;

Results of the biochemical assessment are briefly presented in Table 2 and Figure 2.

**TABLE 2:** Intra‐ and intergroup comparisons of the changes from baseline to the end of the intervention for biochemical parameters

Variable/group	Control		AID	G	GPA	*p* Value^b^
	Mean ± standard deviation					
Inflammatory marker						
High‐sensitivity C‐reactive protein (mg/L)	Before	2.19 ± 0.50	2.68 ± 1.29	2.73 ± 0.85	2.56 ± 0.55	.024
	After	2.03 ± 0.48	1.92 ± 0.97	2.00 ± 0.67	1.30 ± 0.63	<.001
	*p* Value^a^	.07	.002	<.001	<.001	
Blood sugar marker						
Fasting blood sugar (mg/dl)	Before	93.97 ± 12.20	100.55 ± 9.85	98.62 ± 13.1	100.07 ± 9.64	.040
	After	91.97 ± 12.11	95.65 ± 7.13	93.75 ± 12.19	91.9 ± 7.30	.201
	*p* Value^a^	.16	.006	.04	<.001	
Liver enzyme markers						
Alanine transaminase (IU/L)	Before	57.07 ± 6.63	62.58 ± 17.39	62.35 ± 13.58	55.6 ± 11.12	.022
	After	54.35 ± 6.60	58.17 ± 14.99	52.97 ± 10.21	42.32 ± 6.60	<.001
	*p* Value^a^	.3	.1	.0004	<.001	
Aspartate transaminase (IU/L)	Before	37.05 ± 9.30	39.2 ± 12.88	41.72 ± 10.51	54.07 ± 13.89	<.001
	After	37.37 ± 8.48	37.77 ± 12.69	39.05 ± 9.24	42.17 ± 9.79	.144
	*p* Value^a^	.5	.3	.1	<.001	
Alkaline phosphatase (IU/L)	Before	626.4 ± 309.68	661 ± 250.82	679.6 ± 238.92	660.92 ± 162.29	.151
	After	581 ± 292.48	575.57 ± 183.26	510.6 ± 226.49	421.92 ± 136.17	.012
	*p* Value^a^	.2	.04	.0009	<.001	
Serum lipid parameters						
Total cholesterol (mg/dl)	Before	169 ± 37.97	181.37 ± 42.92	165.32 ± 43.27	184.77 ± 45.60	.005
	After	164.3 ± 38.38	168.67 ± 38.17	134.77 ± 29.84	150.81 ± 32.13	<.001
	*p* Value^a^	.3	.08	.0002	.0001	
Triglycerides (mg/dl)	Before	146 ± 41.83	151.65 ± 45.78	155.82 ± 39.60	148.47 ± 45.86	.980
	After	143.45 ± 49.64	145.72 ± 45.07	145.47 ± 32.20	135.35 ± 40	.876
	*p* Value^a^	.4	.3	.1	.08	
Low‐density lipoprotein‐cholesterol (mg/dl)	Before	101.65 ± 22.67	101.97 ± 27.07	105.15 ± 18.22	105.42 ± 18.35	.914
	After	99.72 ± 23.38	97.9 ± 26.35	86 ± 19.68	82.45 ± 14.15	.001
	*p* Value^a^	.3	.3	<.001	<.001	
High‐density lipoprotein‐cholesterol (mg/dl)	Before	43.17 ± 4.75	42.45 ± 5.49	43.32 ± 4.37	41.97 ± 4.83	.570
	After	44.2 ± 5.05	43.72 ± 4.35	44.72 ± 3.63	43.72 ± 3.88	.707
	*p* Value^a^	.1	.1	.06	.03	

*Note*: Data are tested by ^a^ *t*‐test (intragroup) and ^b^one‐way analysis of variance (intergroups).

Abbreviations: AID, anti‐inflammatory diet; G, ginger; GPA, ginger plus anti‐inflammatory diet.

##### Inflammatory markers

At the end of the study, serum CRP‐hs in the intervention groups were significantly reduced compared with the control group (*p* < .001). In addition, the intragroup comparison showed that at the end of the study, compared with the beginning, the serum CRP‐hs in groups AID (*p* = .002), G (*p* < .001), and GPA (*p* < .001) had a significant decrease.

##### Fasting blood sugar

There were no significant differences in serum FBS between groups at the end of the study. However, intragroup comparison showed that at the end of the study, compared with the beginning, serum FBS concentration had a significant reduction in groups AID (*p* = .006), G (*p* = .04), and GPA (*p* < .001).

##### Liver enzymes

At the end of the study, serum ALT concentration in the intervention groups significantly decreased compared with that in the control group (*p* < .001). Further, the intragroup comparison showed that at the end of the study, compared with the beginning, serum ALT concentration had a significant decrease in groups G (*p* = .004) and GPA (*p* < .001). In addition, serum ALP concentration in intervention groups significantly reduced compared with that in the control group (*p* = .01). The intragroup comparison showed a significant reduction in groups G (*p* = .04) and GPA (*p* < .001). Besides, there was no significant difference in serum AST concentration between the intervention groups and the control group at the end of the study. However, the intragroup comparison showed that at the end of the study, compared with the beginning, the serum AST level in the GPA group (*p* < .001) had a significant reduction.

##### Lipid profile assessment

At the end of the study, serum TC concentration in intervention groups significantly decreased compared with that in the control group (*p* < .001). In addition, the intergroup comparison showed that serum TC concentration in groups G (*p* = .0002) and GPA (*p* = .0001) had a significant reduction at the end of the study. Besides, serum LDL‐cholesterol (LDL‐c) concentration in intervention groups significantly reduced compared with the control group (*p* = .001), and the intragroup comparison showed that the serum LDL‐c concentration in groups G and GPA significantly decreased (*p* < .001). Besides, there was no significant difference in serum HDL‐cholesterol (HDL‐c) concentration between the intervention and control groups at the end of the study. However, the intragroup comparison showed that serum HDL‐c concentration in the GPA group (*p* = .03) significantly decreased, whereas the serum TG concentration was not significantly reduced in each intervention group.

##### Fatty liver status assessment

**FIGURE 3:**
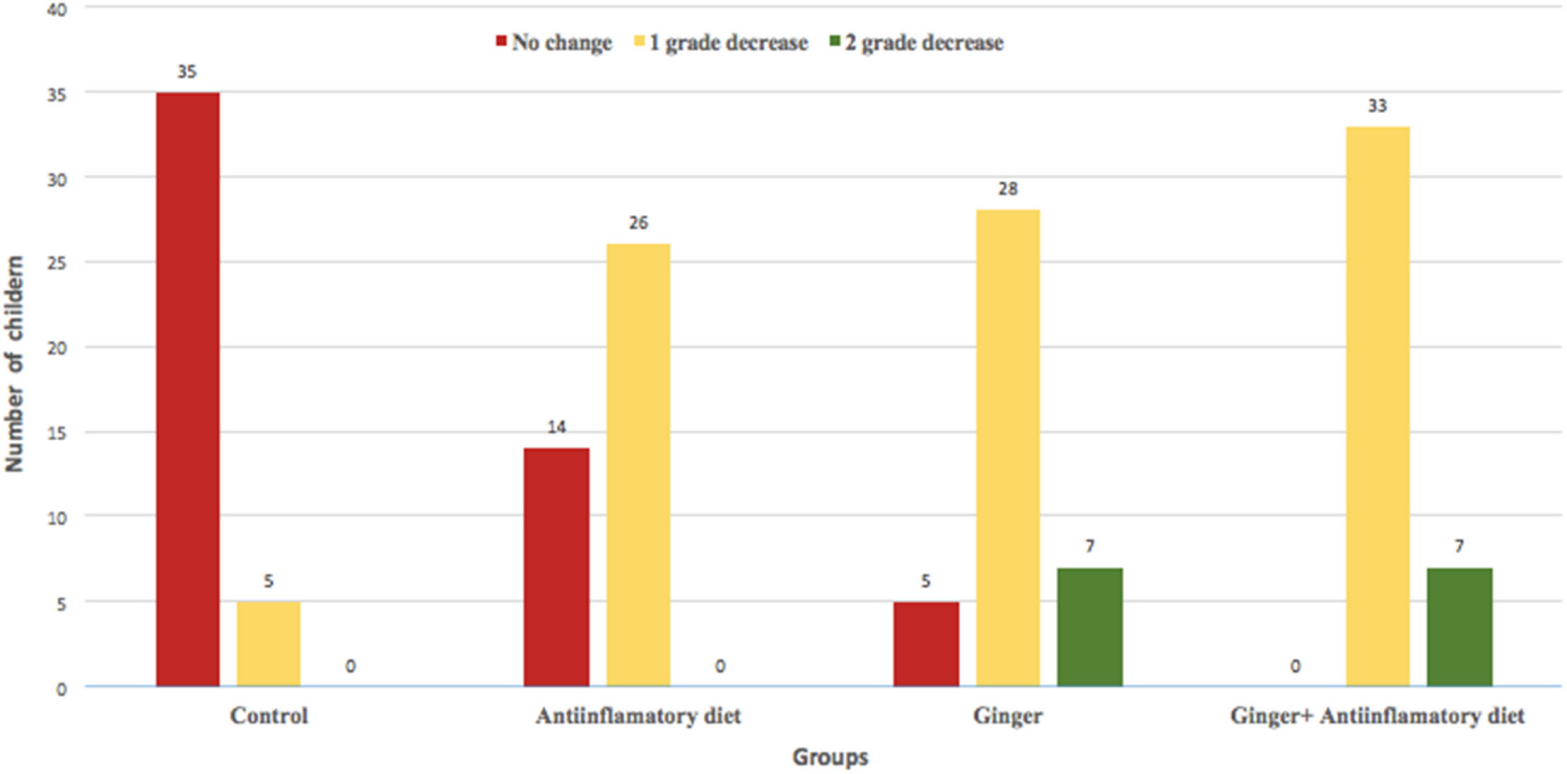
Liver fat status after the study

The results showed a significant reduction in liver fat accumulation in the G and GPA groups at the end of the study. This reduction was higher in the GPA group (one‐degree reduction: 82.5%; and 2‐degree reduction: 17.5%; Figure 3).

### 3.4 Secondary outcome

#### 3.4.1 Anthropometric assessment

**FIGURE 4:**
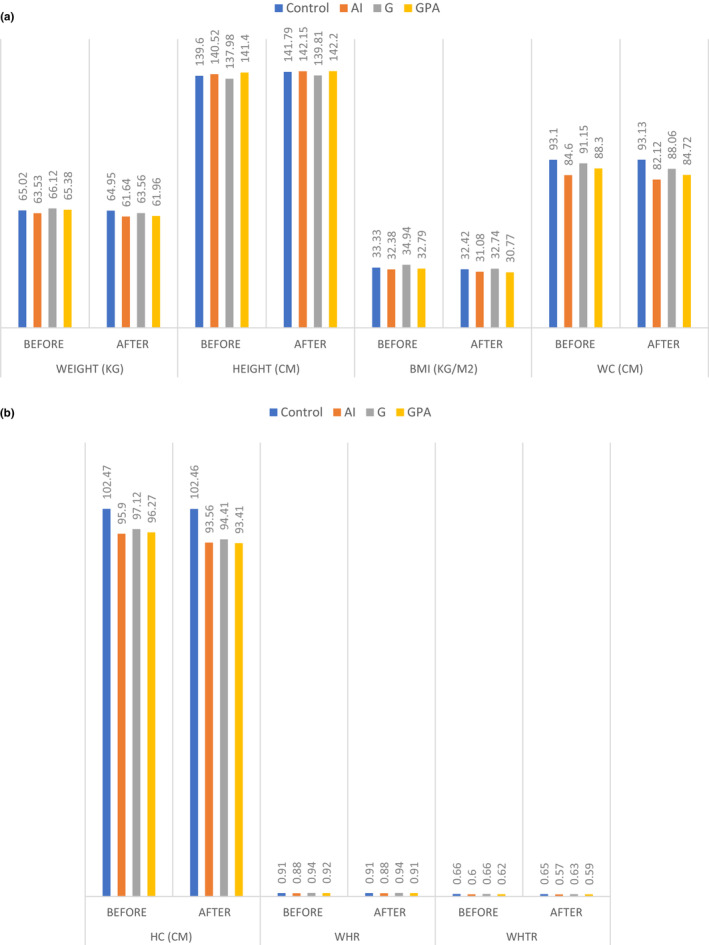
(a) Intra‐ and intergroup comparisons of the changes from baseline to the end of the intervention for anthropometric variables in the four groups. (b) Intra‐ and intergroup comparisons of the changes from baseline to the end of the intervention for anthropometric variables in the four groups. AID, anti‐inflammatory diet; BMI, body mass index; G, ginger; GPA, ginger plus anti‐inflammatory diet; HC, hip circumference; WC, waist circumference; WHR, waist‐to‐hip ratio; WHtR, waist‐to‐height ratio.

**FIGURE 5:**
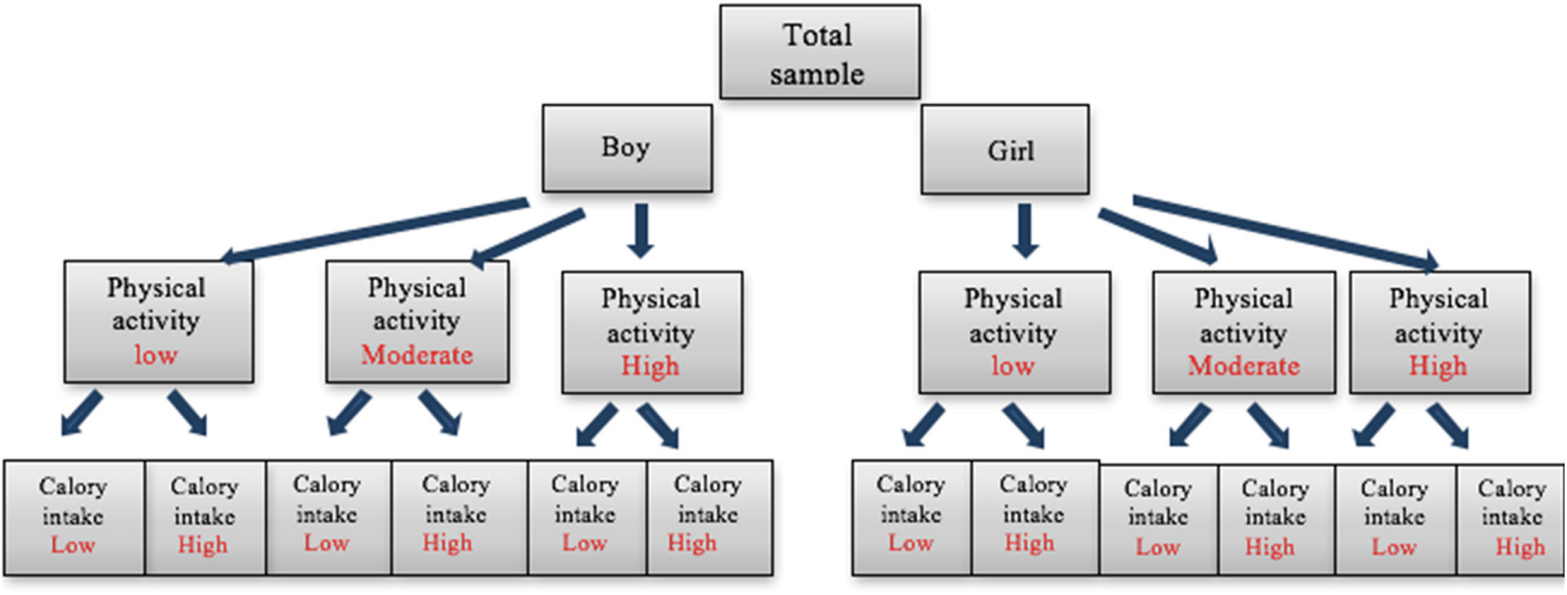
Stratification of patients

Table 3, Figures 4a,b and 5 show inter‐ and intragroups differences, such as intervention and control groups. There was no significant difference in anthropometric indices before and after the intervention in the control and AID intervention groups. A significant decrease in BMI was observed in groups G (*p* = .04) and GPA (*p* = .04) at the end of the study compared with the beginning, but this difference was not significant between groups. Finally, there was a significant difference between WC and WHtR in groups G (*p* = .009 and *p* = .02, respectively) and GPA (*p* = .003 and *p* = .005, respectively). This difference between groups was also significant (*p* < .001).

**TABLE 3:** Intra‐ and intergroup comparisons of the changes from baseline to the end of the intervention for anthropometric variables in the four groups

Variable/group		Control	AID	G	GPA	*p* Value^b^
		Mean ± standard deviation				
Weight (kg)	Before	65.02 ± 10.66	63.53 ± 9.23	66.12 ± 9.34	65.38 ± 9.48	.679
	After	64.95 ± 10.35	61.64 ± 9.36	63.56 ± 9.1	61.96 ± 9.81	.520
	*p* Value^a^	.4	.3	.1	.05	
Height (cm)	Before	139.6 ± 7.23	140.52 ± 8.34	137.98 ± 7.01	141.40 ± 6.90	.188
	After	141.79 ± 7.25	142.15 ± 8.12	139.81 ± 6.70	142.20 ± 6.82	.404
	*p* Value^a^	.49	.47	.48	.4	
Body mass index (kg/m^2^)	Before	33.33 ± 5.55	32.38 ± 5.56	34.94 ± 5.97	32.79 ± 5.30	.191
	After	32.42 ± 5.45	31.08 ± 5.35	32.74 ± 5.82	30.77 ± 5.37	.293
	*p* Value^a^	.2	.1	.04	.04	
Waist circumference (cm)	Before	93.1 ± 6.80	84.6 ± 7.80	91.15 ± 6.84	88.3 ± 5.83	<.001
	After	93.13 ± 6.69	82.12 ± 8.03	88.06 ± 7.56	84.72 ± 5.84	<.001
	*p* Value^a^	.5	.1	.009	.003	
Hip circumference (cm)	Before	102.47 ± 6.93	95.9 ± 8.79	97.12 ± 8.20	96.27 ± 8.89	.001
	After	102.46 ± 6.71	93.56 ± 8.53	94.41 ± 8.09	93.41 ± 8.67	<.001
	*p* Value^a^	.4	.1	.07	.07	
Waist‐to‐hip ratio	Before	0.91 ± 0.081	0.88 ± 0.95	0.94 ± 0.10	0.92 ± 0.10	.03
	After	0.91 ± 0.07	0.88 ± 0.09	0.94 ± 0.11	0.91 ± 0.10	.027
	*p* Value^a^	.5	.5	.5	.3	
Waist‐to‐height ratio	Before	0.66 ± 0.06	0.60 ± 0.06	0.66 ± 0.05	0.62 ± 0.04	<.001
	After	0.65 ± 0.06	0.57 ± 0.06	0.63 ± 0.05	0.59 ± 0.04	<.001
	*p* Value^a^	.2	.07	.02	.005	

*Note*: Data are tested by ^a^ *t*‐test (intragroup) and ^b^one‐way analysis of variance (intergroups).

Abbreviations: AID, anti‐inflammatory diet; G, ginger; GPA, ginger plus anti‐inflammatory diet.

#### 3.4.2 Nutritional assessment

Tables 4 and 5 show the macro‐ and micronutrient intake within and between groups, such as intervention and control groups. At the beginning of the study (week 0), there was no significant difference in food intake within the groups. At the end of the study (week 12), protein intake in the intervention groups significantly decreased compared with that in the control group (*p* = .01). Besides, the intragroup comparison showed that protein intake increased significantly in groups AID (*p* = .02) and GPA (*p* = .02), compared with that in the beginning of the study.

**TABLE 4:** Intra‐ and intergroup comparisons of the changes from baseline to the end of the intervention for nutritional variables in the four groups

Variable/group		Control	AID	G	GPA	*p* Value^b^
		Mean ± standard deviation				
Energy (Kcal/day)	Before	2826 ± 661	2871 ± 639	658 ± 2792	2793 ± 673	.20
	After	2794 ± 652	2756 ± 661	2760 ± 660	2709.6 ± 639	.66
	*p* Value^a^	.73	.33	.73	.92	
Protein (%)	Before	13.4 ± 1.5	13 ± 1.4	13.3 ± 1.9	13.3 ± 1	.85
	After	13 ± 1.6	15 ± 1.4	14 ± 1.6	15.4 ± 1.6	.01
	*p* Value^a^	.20	.02	.17	.02	
Cholesterol (%)	Before	45.5 ± 3.8	45.1 ± 3.8	45.2 ± 3.6	45.18 ± 3.5	.64
	After	46 ± 3.1	45.2 ± 3.6	45.1 ± 3.1	45.44 ± 3.09	.15
	*p* Value^a^	.17	.85	.92	.84	
Fat (%)	Before	41.1 ± 3.3	41.9 ± 3.5	41.5 ± 3.3	41.52 ± 3	.46
	After	41 ± 3.9	39.8 ± 3.3	40.9 ± 3.1	39.16 ± 3.3	.01
	*p* Value^a^	.69	.03	.38	.03	
Water intake (ml/day)	Before	406 ± 189	374 ± 172	387 ± 184	406 ± 236	.12
	After	576 ± 366	554 ± 305	561 ± 298	562 ± 224	.22
	*p* Value^a^	.12	.07	.42	.46	

*Note*: Data are tested by ^a^ *t*‐test (intragroup) and ^b^one‐way analysis of variance (intergroups).

Abbreviations: AID, anti‐inflammatory diet; G, ginger; GPA, ginger plus anti‐inflammatory diet.

**TABLE 5:** Intra‐ and intergroup comparisons of the changes from baseline to the end of the intervention for nutritional variables in the four groups

Variable/group		Control	AID	G	GPA	*p* Value^b^
		Mean ± standard deviation				
Vitamin C (mg)	Before	125.8 ± 62.1	121.6 ± 62.5	129.4 ± 63.4	127.9 ± 65.2	.889
	After	126 ± 63.3	124.7 ± 61.4	127.6 ± 62.8	131.6 ± 63.2	.779
	*p* Value^a^	.98	.02	.84	.02	
Vitamin E (mg)	Before	10.13 ± 16	10.20 ± 16	9.92 ± 15	9.90 ± 14	.735
	After	10.20 ± 16	11.30 ± 11	10.20 ± 16	11.21 ± 11	.834
	*p* Value^a^	.3	<.001	.1	<.001	
Beta‐carotene (mg)	Before	1.579 ± 1.3	1.280 ± 1.3	1.515 ± 1.2	1.345 ± 1.4	.397
	After	1.610 ± 1.4	1.658 ± 1.2	1.420 ± 1.3	1.770 ± 1.3	.041
	*p* Value^a^	.87	.03	.59	.01	
Selenium (mcg)	Before	35.7 ± 10.2	39.4 ± 10.6	41.2 ± 10.1	37.3 ± 10.2	.039
	After	34.9 ± 10.3	44.3 ± 10.1	43.1 ± 10.4	43.6 ± 10.5	.223
	*p* Value^a^	.58	.001	.19	<.001	
Zinc (mg)	Before	10.4 ± 3.3	9.6 ± 3.2	10.2 ± 3.1	9.1 ± 3	.569
	After	10.1 ± 3.3	9.8 ± 3.2	10.6 ± 3.4	9.7 ± 3.3	.402
	*p* Value^a^	.52	.6	.38	.18	

*Note*: Data are tested by ^a^ *t*‐test (intragroup) and ^b^one‐way analysis of variance (intergroups).

Abbreviations: AID, anti‐inflammatory diet; G, ginger; GPA, ginger plus anti‐inflammatory diet.

By contrast, the intragroup comparison showed that fat intake in these two interventions groups decreased compared with that in the beginning of the study (*p* = .03). Besides, fat intake in the intervention groups significantly decreased compared with that in the control group (*p* = .01). It should be noted that there was no significant change in total energy intake within and between groups.

At the end of study, compared with the beginning, dietary intake in vitamin C, vitamin E, beta‐carotene, and selenium increased significantly in groups AI (*p* = .02, <.001, .03, and .001, respectively) and GPA (*p* = .02, <.001, .01, and <.001, respectively), but there was no significant change in zinc intake in any of the groups.

## 4 DISCUSSION

To our knowledge, this is the first double‐blind randomized clinical trial investigating the combined effect of ginger and AID in children with obesity with NAFLD. In this study, daily consumption of ginger in addition to an AID resulted in a significant decrease in FBS, inflammatory marker levels, liver enzymes, dyslipidemia, and liver steatosis. Some human and animal studies have reported multiple pharmacological effects for ginger including inducing weight loss, anti‐inflammatory effects, playing an antioxidant role, lipid metabolism, and improved insulin sensitivity (Ebrahimzadeh Attari et al., 2017). Gingerol and shogaol are considered bioactive compounds in fresh and dried ginger, respectively, and are responsible for many of the pharmacological properties of ginger (Suman et al., 2021). Recently, the beneficial effects of ginger on weight control have been studied. The exact mechanisms of ginger's effect on weight loss are not fully understood. Several possible mechanisms were suggested, including suppression of pancreatic lipase, decreasing intestinal fat absorption, increasing adipose tissue lipolysis by increasing activation of hormone‐sensitive lipase, suppression of lipogenesis and lipid accumulation by decreasing induction of fatty acid synthesis, increasing thermogenesis, and appetite controlling (Atashak et al., 2011). In this study, BMI, WC, and WtHR decreased significantly in the G and GPA groups. Few human studies have examined the effect of ginger on anthropometric indices, and the results of these studies are contradictory. Ebrahimzadeh et al., had studied the effect of ginger supplementation on women with obesity. The results of their study showed significant reduction in BMI compared with placebo (Attari et al., 2016), which was consistent with our study results. In contrast to our study, in Atashak et al.'s study ginger supplementation (1 g per day) in men with obesity (*n* = 8) did not show a significant difference in body composition and anthropometric indices between ginger and control groups. Further, in the study by Rafie et al. (2020), no significant difference in body composition and anthropometric indices were observed with ginger supplement (1.5 g per day).

Most animal studies have supported ginger's effectiveness in weight loss in animal models. By contrast, human studies show that anthropometric properties change little or not at all (Ebrahimzadeh Attari et al., 2017). However, it must be taken into account that in most animal studies, ginger extract or its active ingredients were used instead of ginger powder, which may justify this difference between the results of animal and human studies (Ruiz‐Canela et al., 2015). Nevertheless, regarding the effectiveness of an AID on NAFLD, no significant changes in body composition and anthropometric indices were observed in the AID group. Our findings are consistent with an observational study on 430 young Americans aged 21–35 years (Wirth et al., 2016) and the study by Aslani et al. (2019), which indicated no significant association between DII and BMI.

Our study showed a significant reduction of FBS in all three intervention groups compared with the control group. Similar to our study, Rafie et al. (2020) reported a significant decrease in FBS with daily consumption of ginger. Similarly, the study by Rahimlou et al. (2016) showed that consuming ginger reduced insulin and FBS. In this regard, Mozaffari‐Khosravi et al. (2014) reported that ginger supplementation caused a meaningful decrease in FBS and hemoglobin A_1_C. Findings from similar animal studies confirm these results. For this effect, ginger inhibits the liver phosphorylase enzyme to prohibit degradation of glycogen stored in liver cells. By inhibiting glucose 6‐phosphatase activity, ginger supplementation can reduce the degradation of glucose 6‐phosphate into glucose, thereby reducing blood glucose (Nammi, Sreemantula & Roufogalis, 2009; Shanmugam et al., 2011). Ginger also appears to reduce glucose uptake by inhibiting enzymes in glucose metabolism, such as α‐glucosidase and amylase in the gut. This antioxidant compound may increase the expression of GLUT4 (glucose transporter type 4) protein insulin receptors, and improve the function of β‐pancreatic cells, thereby improving glucose tolerance (Li et al., 2012). The results of this study are also consistent with previous studies on AIDs. The study by Nikbakht‐Jam et al. (2016) demonstrated a positive effect of an AID on lowering blood sugar in patients with diabetes.

Another finding of our study was the antilipidemic effect of ginger. Our results indicated that daily consumption of ginger compared with placebo significantly reduced LDL‐c and TC levels and increased HDL‐c level when consumed with an AID. However, serum TG levels did not change in any of the intervention groups. The results of previous clinical trials that investigated the effect of ginger on lipid profiles are, however, contradictory. In the study by Rahimlou et al. (2016) an intervention using ginger reduced serum TG, whereas Alizadeh‐Navaei et al. (2008) reported that an intervention with 3 g/day of ginger in patients with hyperlipidemia significantly reduced serum TG and TC levels compared with the control. Mozaffari‐Khosravi et al. (2014) observed that daily consumption of 3 g of ginger by patients with diabetes leads to a decrease in the serum LDL‐c level, but does not affect the TC, TG, and HDL‐c levels. The present study's findings on the effectiveness of ginger on serum cholesterol concentration are consistent with those of many animal studies (Nammi, Sreemantula & Roufogalis, 2009; ElRokh et al., 2010). Cholesterol reduction by ginger can be explained as follows: ginger can increase the conversion of cholesterol in to bile acids by increasing the activity of cholesterol‐7α‐hydroxylase, leading to a decrease in serum cholesterol levels (Alizadeh‐Navaei et al., 2008). Biocompounds in ginger have also been reported to suppress cholesterol synthesis in rat liver cells (Bordia, Verma & Srivastava., 1997). The findings of this study regarding the lack of effect of ginger supplement on serum HDL‐c are consistent with those of some previous studies (Rafie et al., 2020; Mozaffari‐Khosravi et al., 2014; Alizadeh‐Navaei et al., 2008 & Bordia, Verma & Srivastava., 1997). This is probably because changes in HDL‐c levels are less affected by dietary components.

This study showed that ginger supplementation plus an AID can reduce serum hs‐CRP levels. The hs‐CRP is mainly produced in the liver, and its serum level is associated with the rate of liver inflammation. In the study by Arablou et al. (2014), a daily intake of ginger (1.6 g) significantly reduced serum hs‐CRP level in patients with diabetes. In addition, a study by Atashak et al. (2012) showed that ginger powder could reduce serum hs‐CRP concentration in men with obesity, which is similar to the results of our study. It seems that the efficacy of ginger on inflammation is due to the positive effect of some active ingredients (gingerol and zerumbone) in inhibiting NF‐κB (nuclear factor kappa B) and TNF‐α (tumor necrosis factor alpha). Ginger inhibits NF‐κB activity by inhibiting the *TNF‐α* gene, and thus inhibits the production of acute‐phase positive proteins such as CRP (Kim et al., 2004). By contrast, the present study contradicts the findings by Hart et al. (2021), who examined the effect of an AID on serum CRP. In a cross‐sectional study of adolescents aged 10–17, Yilmaz et al. (2012) reported decreased CRP adherence to an AID.

Our findings showed that consumption of ginger compared with control resulted in a reduction in serum ALT and ALP concentrations. However, there was no significant change in AST levels. These findings were consistent with the findings of a previous study. Rahimlou et al. (2016) showed that ginger supplementation significantly reduced serum ALT and gamma‐glutamyl transferase (GGT) levels compared with placebo. Further, these results were also observed by Rafie et al. (2020). The effect of ginger on liver fibrosis in rats showed that treatment with a ginger extract induced a significant reduction in AST and ALT levels as well as a protective effect against liver fibrosis. Although the exact mechanism of this protective effect of ginger is not yet fully understood, insulin resistance is one of the reasons for elevated liver enzymes. Insulin resistance activates lipolysis in adipose tissue and increases the free flow of fatty acids to the liver, leading to steatosis and liver damage. In this regard, Pagano et al., reported that ginger supplementation reduces the indicators related to insulin resistance and improves liver enzymes (Pagano et al., 2021).

In the AID group, there was a significant decrease in ALP levels. Darbandi et al. (2020) have shown that adherence to an AID is associated with lower liver enzymes, including AST, ALT, and GGT. It seems that weight loss inhibits the accumulation of lipid in hepatocytes, resulting in a reduction of inflammatory response via NF‐κB activation and cytokine production, which leads to a decrease in liver damage. Lack of a significant decrease in liver enzymes in the present study may be due to the low sample size.

Finally, the main finding of this study was a significant reduction in liver steatosis in groups G and GPA. It was also observed that this decrease in hepatic steatosis in the groups receiving ginger was increasingly more significant than the AID group. Results of this study were, however, inconsistent with the studies by Rafie et al. (2020) and Rahimlou et al. (2016). Animal studies have also shown that the active compounds in ginger can reduce fatty liver disease (Liu et al., 2013). One of the main reasons for this protective property is the activity of ginger on the expression of the proliferating cell nuclear antigen (PCNA). PCNA is a nuclear protein involved in the regulation of cell proliferation. In liver diseases, such as NAFLD and cirrhosis, PCNA expression is increased, leading to excessive and uncontrolled proliferation of damaged tissue to replace damaged tissue and liver fibrosis. Previous studies have shown that ginger supplementation can reduce PCNA expression and prevent the intensification of liver fibrosis (Abdulaziz Bardi et al., 2013). Furthermore, in this area, Tyrovolas et al. (2019) conducted a prospective study to investigate the effect of an AID on 3042 individuals with NAFLD, which showed that following an AID, fat accumulation is reduced which can subsequently prevent NAFLD. In general, visceral adipose tissue can stimulate more lipolysis and the excretion of free fatty acids into the bloodstream, which contributes to the more significant accumulation of TGs in the liver. The mechanisms responsible for reducing liver fat following the use of ginger supplements are probably a change in energy supply, increased oxidation of fats in liver cells, burning and further metabolism of visceral fat stores. Thus, when following an AID in combination with ginger supplementation, this positive regulation likely becomes more tangible (Nayebi Far & Ghasemi, 2021).

Finally, based on the between‐intervention groups comparison in this study, it was concluded that ginger (G group) is more effective than the AID (AID group) in the treatment of fatty liver in children. By contrast, taking an AID and ginger together (GPA group) is even more effective than taking ginger alone. Therefore, it can be concluded that ginger as a nonmedicinal supplement can improve fatty liver in children with obesity, while adherence to an AID will increase its effectiveness.

The strength of this study was that it is the first to evaluate the effect of two interventions alone and together on pediatric fatty liver. However, there are some limitations, such as the small sample size and lack of complete adherence to diet by some participants. Nevertheless, it should be noted that these are not weaknesses just for our study. Still, it is one of the limitations of lifestyle‐modification studies, because according to the results of published studies, only 30% of patients participating in lifestyle‐modification programs for the treatment of NAFLD were able to achieve the desired weight loss (>5%) at the end of 1 year (Vilar‐Gomez et al., 2015).

## FUNDING INFORMATION

This study was approved and supported by a grant from Kermanshah University of Medical Sciences, Kermanshah, Iran (grant number 990165).

## CONFLICTS OF INTEREST

The authors declare no conflict of interest.

## ETHICS STATEMENT

The Medical Ethics Committee of Kermanshah University of Medical Sciences has approved the implementation of this research (IR.KUMS.REC.1399.085). Moreover, it has been recorded in the Iranian Clinical Trial Registry (IRCT20181111041611N3) and is available through www.irct.ir. All participants signed a written informed consent form.

## Data Availability

The data sets used and/or analyzed during this study are available from the corresponding author on reasonable request and permission for use was received by the Ethics Committee of Kermanshah University of Medical Sciences.
